# Perception and Attitude of People Toward Onchocerciasis (River Blindness) in South Western Nigeria

**DOI:** 10.4103/0974-9233.71594

**Published:** 2010

**Authors:** A. O. Adeoye, A. O. Ashaye, O. H. Onakpoya

**Affiliations:** Ophthalmology Unit, Obafemi Awolowo University Teaching Hospital, Ile-Ife, Nigeria; 1 Ophthalmology Department, University College Hospital, Ibadan, Nigeria

**Keywords:** Attitude, Onchocerciasis, Perception, River Blindness

## Abstract

**Background:**

Onchocerciasis (river blindness) is a major cause of bilateral blindness with devastating socioeconomic consequences. Since Nigeria is the most heavily onchocerciasis endemic country in the world, the information on people’s knowledge about this disease is significant. This could influence their response to current preventive measures of the African Programme for Onchocerciasis Control.

**Aim:**

This study was designed to estimate the level of knowledge and attitudes of rural/semi-urban communities in Ife North Local Government Area of Osun State toward onchocerciasis.

**Materials and Methods:**

Cluster random sampling was used to select 500 adults for the study. Semi-structured questionnaires were administered to subjects. Data on knowledge of the local name, cause, mode of transmission, manifestation, severity, treatment, and prevention of onchocerciasis were collected and analysed. Statistical analysis included frequency distribution of the responses and a Chi-square test for comparison of variables with the *P* value for statistical significance set at 0.05.

**Results:**

Onchocerciasis was well known by its local name among 458 (91.6%) of the respondents. Only seven (1.4%) knew that it affects both the eyes and skin. The cause was commonly attributed to impure blood by 114 (22.8%), whereas transmission was thought to be through fomites by 161 (32.2%). Only 12 (2.4%) respondents attributed the disease to blackfly bites. The level of education and the association of onchocerciasis with a river were significantly associated (*P* = 0.001). Subcutaneous nodules were felt to contain water (85.4%), baby worms (3.2%), and fat (0.6%). There was a negative attitude toward sufferers of the disease.

**Conclusion:**

Adequate information transfer in simple local dialect by trained personnel to the communities at risk of onchocerciasis is essential for better uptake of all aspects of the onchocerciasis control programme.

## INTRODUCTION

Onchocerciasis or river blindness is a chronic parasitic disease caused by the filarial nematode worm *Onchocerca volvulus* and transmitted by blackfly bites. Onchocerciasis is most prevalent in Africa, where over 99% of the cases occur. Onchocerciasis occurs in Nigeria with greater frequency than any other country worldwide.^1^ Considered endemic, 40% of all cases in Africa occur in Nigeria.^1^ It is one of the priority diseases of the VISION 2020 initiative for eliminating avoidable blindness.^2^

Onchocerciasis causes generalized itching, thickening and depigmentation of skin, subcutaneous nodules, and visual impairment which may progress to complete blindness, with grave socioeconomic consequences. Interaction with patients has shown that many lacked understanding and knowledge of this blinding condition. Studies conducted in developed countries show that patients have elaborate and sophisticated theories about their illnesses.^3^ In developing countries such as Nigeria, there are a complex set of beliefs and values associated with health and illness.^4^,^5^ People’s attitude to a disease process, manifestation, treatment, and various aspects of prevention are influenced by their knowledge and perception of the condition. In a country with a significant prevalence of onchocerciasis, it is pertinent from the public health policy perspective that such beliefs and attitudes need to be explored. This prompted us to further examine the issue of onchocerciasis on a wider scope, especially as the ailment is still the second common cause of blindness in Nigeria.^6^

The aim of this study was to examine the perception and attitude of onchocerciasis in the local populus living in rural and semi-urban communities of Ife North local government area of southwestern Nigeria. This data would provide useful baseline data for appropriate intervention and public health policy.

## MATERIALS AND METHODS

This study was conducted in November 2004 in Akinlalu-Ashipa Ward of Ife North local Government area of Osun State, southwestern Nigeria. Ethics approval was received from the Research and Ethics Committee of Obafemi Awolowo University Teaching Hospital (OAUTH), Ile-Ife.

The ward is situated west of Ile-Ife town and consists of two semi-urban towns and 38 villages, many of which are in close proximity to the fast-flowing River Opa. The tropical climate combined with the fast moving water is conducive for breeding of vectors such as the black fly of the genus *Simulium*.^7^ The local population receives its water supply mainly from rivers and a few deep wells. Local medical services consist of one dispensary and one maternity ward in each of the larger towns. There are neither mobile nor local eye care services for 50–100 km, and many of the residents of this region have to travel to OAUTH, Ile-Ife for ophthalmic services.

A multistage cluster random sampling technique was used to select 500 adult subjects from the 12 villages where a survey of blindness had previously been conducted and reported elsewhere.^6^ Informed verbal consent was obtained with the assistance of village heads. All households within the boundaries of selected clusters were visited to identify adults aged 16 years or older who were permanent residents (i.e., residents for at least 1 year). Five interviewers were trained to conduct the interviews, and a pilot study was performed administering the questionnaire on 10 primary school teachers in one of the villages to standardize the questionnaire. Results from the pilot study were not included in the main study. Semi-structured questionnaires with open-ended questions were given to respondents after receiving their informed verbal consent. Communication was through the main Yoruba dialect. Data collected included age, sex, occupation, religion, and educational status of respondents. Other details in the questionnaire were knowledge of local name for river blindness, cause, mode of transmission, manifestation, severity, treatment, and prevention of the disease. Responses were entered on the spaces provided in the questionnaire, and data were coded for entry into a computerized database. All data were analysed, using Statistical Package for Social Sciences (Version 10.0, SPSS Inc., Chicago, IL, USA). Statistical methods included frequency distribution of the responses and a Chi-square test for comparison of variables with the P value for statistical significance set at 0.05.

## RESULTS

Of the 500 subjects interviewed, 267 (53.4%) were males and 233 (46.6%) were females. The age of the cohort ranged between 16 and 85 years. The number of subjects in the age group ranging from 26 to 35 years was 29.6%, followed by the 23.2% in the age group 36 to 45 years. The majority of subjects (76.8%) were married and 320 (64%) were Christians, 173 (34.6%) were Muslims, whereas 7 (1.4%) belonged to a traditional religion. Only 63 persons (12.6%) were illiterate, 239 (47.8%) had received secondary education, and 52 (10%) had post-secondary education. Most of the subjects 189 (37.8%) were traders, 94 (18.8%) artisans, 83 (16.6%) farmers, and 39 (7.9%) were civil servants.

The majority of the subjects, 458 (91.6%) knew the local name for river blindness as “Inarun” while other names included “Kuruna” 6 (1.2%), “komo bus stop” 3 (0.6%), and “ifon” 1 (0.2%). Thirty-one subjects (6.2%) did not know the local name of the disease. Ninety-six percent of the illiterate respondents knew the local names compared to 90.8% of educated subjects. Increasing level of education did not indicate local knowledge [Table 1].

**Table 1:** Knowledge of onchocerciasis in adult participants of southwestern Nigeria (2004)

Correct responses about onchocerciasis	Number of respondents	%
Local name	458	91.6
Involvement of eyes and skin	7	1.4
Severe	483	96.6
Skin lesions not infectious	40	8.0
Curable	486	97.2
Disfiguring	459	91.8
Not fatal	84	16.8

Responses regarding the type of disease indicated 358 (71.6%) participants thought onchocerciasis is only a skin infection, 44 (8.8%) believed it was a blood-borne infection. The seven (1.4%) respondents who knew it could affect both skin and eyes were all educated [Table 1]. Causes of onchocerciasis were attributed to impure blood (22.8%), eating food like “garri” (smoked cassava grains), kolanut, groundnut (21.2%), poor hygiene (19.8%), impure water (11.6%), bacterial infection (8.2%), and alcohol intake (2%) [Table 2]. Forty-four subjects (8.8%) had no idea about the cause of the disease [Table 2].

**Table 2:** Responses on the cause of onchocerciasis in southwestern Nigeria (2004)

Cause	Number of respondents	%
Impure blood	114	22.8
Food	106	21.2
Poor hygiene	99	19.8
Impure water	58	11.6
Bacterial infection	41	8.2
Alcohol intake	10	2.0
Fomites	8	1.6
Others	20	4.0
Don’t know	44	8.8
Total	500	100

Transmission of onchocerciasis was thought to be via fomites (32.2%), mosquito bites (17%), sexual intercourse (12.2%), witchcraft (9.2%), heredity (4.2%), and food (3.2%) [Table 3]. Only 12 (2.4%) of respondents answered that transmission was due to a black fly bite [Table 3].

**Table 3:** Responses about the transmission of onchocerciasis in southwestern Nigeria (2004)

Response	Literate	Non-literate	Total	%
Fomites	134	27	161	32.2
Dirty water	75	14	89	17.8
Mosquito	74	11	85	17.0
Sexual intercourse	61	–	61	12.2
Witchcraft	42	4	46	9.2
Mother to child	18	3	21	4.2
Food	14	2	16	3.2
Blackfly	10	2	12	2.4
Don’t know	9	–	9	1.8
Total	437	63	500	100

Significance of a river as medium of transmission was recognized by 168 (33.6%) respondents. Other respondents 132 (26.4%) felt that body contact with water through swimming in the river or stagnant water was the mode of transmission. A total of 161 respondents (32.2%) believed the river was not significant in transmission. There was a statistically significant relationship between the level of education of the respondents and their association of onchocerciasis with a river (*P* = 0.001).

The most common local name for the skin rash was “kuruna” (55.4%). Other names included “inarun” (36.6%) and “ifon” (1.2%). Of all respondents, 4.4% did not know the common local name. The skin lesion was thought to be disfiguring by 481 (96.2%) respondents.

Data on the subcutaneous nodule showed that 427 (85.4%) respondents felt the nodule contained water, 16 (3.2%) believed it contained baby worms and 3 (0.6%) believed it was fat, 6 (1.2%) responded the nodule contained adult worms, and 48 (9.6%) respondents had no idea.

Of the 341 (68.2%) that knew that onchocerciasis could cause blindness, 154 (45.2%) felt it was because blurred vision worsens with age, 62 (18.2%) believed that it was due to excessive scratching of eyes, 22 (6.5%) felt it could result if the infection is overwhelming and 103 (30.2%) did not know how onchocerciasis could cause blindness.

In the cohort 245 (49%) claimed that they or family members had contracted onchocerciasis previously. Treatment of the disease ranged from 76 respondents (31.0%) using traditional treatment (herbs and potions), 34 (13.9%) using western (orthodox) therapy, and 133 (54.3%) combined western and traditional treatment. Two (0.8%) used prayers only. However, out of the 500 respondents, traditional treatment was perceived as the ideal mode of treatment by the majority (41.4%); 164 (32.8%) stressed western treatment, whereas 84 (16.8%) respondents combining the two types of treatment [Table 4].

**Table 4:** Responses on the ideal treatment for onchocerciasis in southwestern Nigeria (2004)

Response	No	%
Traditional herbs and potions Western	207	41.4
(orthodox)	164	32.8
Prayers	6	1.2
Combination	84	16.8
Don’t know	39	7.8
Total	500	100

A total of 455 (91%) respondents were positive that the disease is preventable, out of which 131 (28.8%) claimed the means of prevention was through improving personal hygiene, 119 (26.2%) stressed avoiding contact with infected persons, 59 (13.0%) stressed the use of preventive drugs, 48 (10.5%) stressed the use of preventive herbs, whereas 39 (8.6%) believed in the maintenance of a wide combination of health behaviors ranging from observing good hygiene, use of drugs, and insecticides, to avoidance of sexual intercourse. On the role of the community in the control of onchocerciasis, 134 (26.8%) respondents believed environmental hygiene is essential, 89 (17.8%) believed in health education, 51 (10.2%) stressed the need to support the sick, whereas 48 (9.6%) believed in encouraging the sick to comply with prescribed treatment. Although 174 (34.8%) respondents claimed the community has nothing to do with disease control, 4 (0.8%) wanted the community to prevent infected persons from bathing in the river that is being used by the community for different purposes. Their suggestions on the role of government in controlling the disease ranged from provision of free drugs (79.0% of respondents), health education (8.8% of respondents), provision of good water (3.2% of respondents), environmental hygiene (2.8% of respondents), immunization against onchocerciasis (2.0% of respondents) to government recognition of traditional healers (1.6%).

## DISCUSSION

This study highlights the lack of knowledge of rural and semi-urban communities of southwestern Nigeria regarding fundamental information about onchocerciasis. Most people with the disease live in rural areas beyond the reach of government health services. This scenario is similar to studies in Cameroon^8^ and the Edo State of Nigeria.^9^ The majority of the respondents were aware of the menacing disease, hence its local name “Inarun” (fire from heaven) which depicts the characteristic unsightly hypo-/hyperpigmented skin lesions. Hence, a majority (71.6%) of the respondents attributed the disease to an obvious skin infection. This is similar to the Cameroon study^8^ where 60% recognized “leopard” skin as manifestation of onchocerciasis. Unlike the Cameroon study where 90% were aware that the black fly was responsible for transmission, the level of awareness in our study was low, with only 2.4% of the respondents associating a blackfly bite with onchocerciasis. This is probably due to the greater governmental (political) commitment to control onchocerciasis in Cameroon.

Our results combined with those of previous studies in Nigeria, draw attention to the need for more aggressive advocacy, awareness, and sensitization programmes in rural communities of Nigeria. In this study, there was a low level of knowledge about subcutaneous nodules similar to the Edo State study^9^ where nodules were believed to be blood clots, whereas in Enugu,^10^ only 34% attributed nodules to blackfly bites. This underscores the importance of health education as a means of onchocerciasis control.

Hewlett *et al*. reported that 66% of participants in their study believed onchocerciasis could be transmitted sexually.^8^ This was higher than 42.8% found in our study. Heredity and maternal transmission were prominent in our study and Hewlett *et al*. as people tend to associate these factors with the disease conditions they cannot explain. Our results are consistent with Wagbatsoma and Okojie^11^ who reported that the signs and symptoms of onchocerciasis were given specific local names indicating that they may be separate disease entities.

Odebiyi and Ekong,^12^ had documented the widespread belief in magic and witchcraft in Nigeria. In an earlier community-based study in southwestern Nigeria, Adeoye^6^ found that 74% of blind subjects believed that extraneous forces beyond their control caused blindness. Many people tend to seek help from traditional healers, which may explain why the majority of our cohort considered the traditional mode of treatment, the most ideal. They claimed that traditional medicine is what their ancestors turned to and this is the alternative that was passed on to the subjects in the cohort. The dearth of medical facilities and personnel in remote areas may further substantiate their claim and continue the hold of traditional medicine in these areas as no other alternative is presented.

Traditional healers have long been an integral part of the Yoruba culture, persisting in rural communities where they live and work. Traditional healers represent a strong cultural force with a significant clout that is evidenced by respondents in our cohort, suggesting the government’s recognition of traditional healers. This is consistent with Courtright *et al*.,^13^ who suggested that government may build upon the existing respect given to healers by the community, by organising collaborative eye care programmes with healers, using their pre-existing knowledge and skills. Poverty in rural communities, with inability to purchase pharmaceutical drugs may contribute to the preference for traditional medication. Poverty likely explains why 79% of the cohort stressed that government should supply free drugs as a means of prevention of onchocerciasis. Hence, without proper information on the scientifically tested efficacy of orthodox (western) treatment, the uptake of mass distribution of ivermectin may be limited.

In this study, subjects showed negative attitude toward those affected with onchocerciasis due to the unsightly skin and blindness. These attitudes are similar to those reported by Wagbatsoma and Okojie.^11^ This outcome was expected as the Yoruba believe in the “normal” and “perfect” and abhor the “abnormal” and “imperfect.”^14^ Normal people treat the handicapped as less than normal and whenever possible, avoid sustained social interaction with them. Over a third (32.2%) claimed onchocerciasis transmission is through fomites, and 26.2% believed avoidance of infected persons was the only means of prevention.

We agree with Richards *et al*.^15^ that community acceptance and participation are essential for the success of mass ivermectin chemotherapy programme for onchocerciasis. For this to be possible, adequate transfer of information to communities at risk of onchocerciasis in simple local language, using appropriately trained community health extension workers is necessary. This may be through the mass media in the towns that have electricity; school health services in villages as children can be used to disseminate information to their parents and neighbors. Daily health presentations in health centers and dispensaries should include information on the cause, transmission and signs and symptoms of onchocerciasis, including the promotion of a positive attitude toward sufferers.

In conclusion, there was significant awareness of the severe, curable, and disfiguring nature of onchocerciasis in the community. However, there was a relative paucity of knowledge regarding the cause, transmission, treatment, and care of the infected individuals among southwest Nigerians. This study provides information for the formulation of intervention strategies and public health policies which would be acceptable to communities at risk of onchocerciasis, compatible with their lifestyles and thus sustainable.
